# 7-(2,4-Dichloro­phen­yl)-2-methyl­sulfanyl­pyrazolo[1,5-*a*]pyrimidine-3-carbonitrile

**DOI:** 10.1107/S1600536809014792

**Published:** 2009-04-25

**Authors:** Li-rong Wen, Huai-yuan Xie, Shu-wen Wang

**Affiliations:** a College of Chemistry and Molecular Engineering, Qingdao University of Science and Technology, Qingdao 266042, People’s Republic of China

## Abstract

In the mol­ecule of the title compound, C_14_H_8_Cl_2_N_4_S, all the ring atoms in the pyrazolopyrimidine system are almost coplanar, the largest deviation from the mean plane being 0.027 (2) Å for a C atom. The conformation of the methyl­sulfanyl group is anti­periplanar, with a torsion angle of −176.7 (2)°. A weak inter­molecular C—H⋯N hydrogen bond and a Cl⋯N halogen bond [Cl⋯N = 3.196 (5) Å] with a nearly linear N⋯Cl—C angle [174.2 (1)°] link the mol­ecules into a two-dimensional assembly. Face-to-face π–π stacking, with a centroid–centroid separation of 3.557 (2) Å and an angle of 7.1 (1)° between the two planes, completes the inter­molecular inter­actions in the solid state.

## Related literature

For the biological activity of pyrazolo[1,5-*a*]pyrimidine derivatives, see: Li *et al.* (1995 ▶). For applications of enamino­nes, see: El-Taweei *et al.* (2001 ▶); Hernandez *et al.* (2003 ▶); Olivera *et al.* (2000 ▶). For bond-length data, see: Allen *et al.* (1987 ▶). For Cl⋯N halogen bonds, see: Chu, *et al.* (2001 ▶); Lommerse *et al.* (1996 ▶); Ramasubbu *et al.* (1986 ▶).
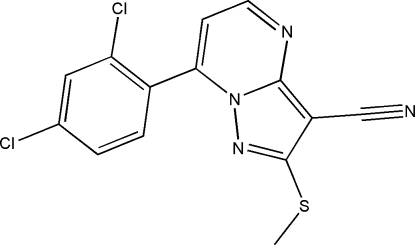


## Experimental

### 

#### Crystal data

C_14_H_8_Cl_2_N_4_S
*M* *_r_* = 335.20
Monoclinic, 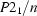

*a* = 8.230 (2) Å
*b* = 14.656 (4) Å
*c* = 12.667 (4) Å
β = 108.460 (5)°
*V* = 1449.3 (7) Å^3^
*Z* = 4
Mo *K*α radiation
μ = 0.59 mm^−1^
*T* = 293 K
0.32 × 0.26 × 0.22 mm

#### Data collection

Bruker SMART CCD area-detector diffractometer
Absorption correction: multi-scan (*SADABS*; Sheldrick, 1996 ▶) *T*_min_ = 0.814, *T*_max_ = 0.879
8252 measured reflections
2965 independent reflections
2181 reflections with *I* > 2σ(*I*)
*R*_int_ = 0.032

#### Refinement

*R*[*F* ^2^ > 2σ(*F* ^2^)] = 0.043
*wR*(*F* ^2^) = 0.095
*S* = 1.04
2965 reflections
191 parameters
H-atom parameters constrained
Δρ_max_ = 0.22 e Å^−3^
Δρ_min_ = −0.29 e Å^−3^

### 

Data collection: *SMART* (Bruker, 1998 ▶); cell refinement: *SAINT* (Bruker, 1999 ▶); data reduction: *SAINT*; program(s) used to solve structure: *SHELXS97* (Sheldrick, 2008 ▶); program(s) used to refine structure: *SHELXL97* (Sheldrick, 2008 ▶); molecular graphics: *SHELXTL* (Sheldrick, 2008 ▶); software used to prepare material for publication: *SHELXTL*.

## Supplementary Material

Crystal structure: contains datablocks global, I. DOI: 10.1107/S1600536809014792/zl2192sup1.cif

Structure factors: contains datablocks I. DOI: 10.1107/S1600536809014792/zl2192Isup2.hkl

Additional supplementary materials:  crystallographic information; 3D view; checkCIF report

## Figures and Tables

**Table 1:** Hydrogen-bond geometry (Å, °)

*D*—H⋯*A*	*D*—H	H⋯*A*	*D*⋯*A*	*D*—H⋯*A*
C8—H8⋯N4^i^	0.93	2.61	3.474 (3)	154

Symmetry code: (i) 
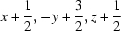
.

**Fig. 1.**
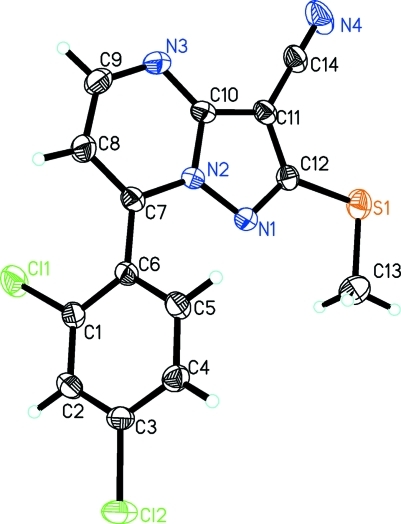
View of the title compound with 35% probability ellipsoids.

**Fig. 2.**
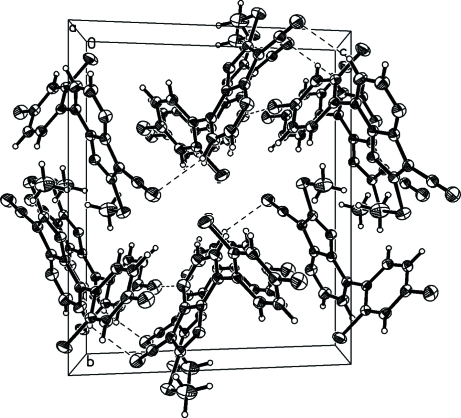
The molecular packing of the title compound viewed along the a axis. Dashed lines indicate the hydrogen bonds and N···Cl short contacts.

**Fig. 3.**
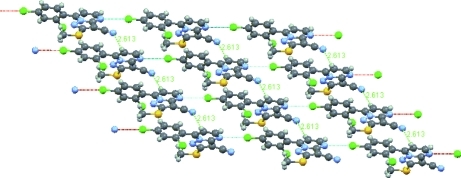
Diagram of two-dimensional structure linked by the hydrogen bonds and N···Cl short contacts.

## supplementary crystallographic information

### Comment

Pyrazolo[1,5-*a*]pyrimidine derivatives have been reported to show various biological activities such as antibacterial, insulin releasing, anti-inflammatory activities (Li *et al.*, 1995). Enaminones have been widely used as building blocks in the synthesis of pyrazolo[1,5-*a*]pyrimidine derivatives (El-Taweei *et al.*, 2001; Hernandez *et al.*, 2003; Olivera *et al.*, 2000). We report here the crystal structure of title compound (Fig.1), which was synthesized by reaction of 1-(2,4-dichlorophenyl)-3-dimethylamino-2-en-1-one and 3-methylsulfanyl-4-cyano-5-amino-1*H*-pyrazole in the presence of acetic acid.

The bond lengths and angles in this compound are within normal ranges (Allen, 2002). All the ring atoms in the pyrazolopyrimidine moiety are almost coplanar, the largest deviation from the mean plane being 0.027 (2)Å for atom C10. The dihedral angle between the pyrazolopyrimidine moiety and the benzene ring is 54.9 (5)°. The conformation of the methylsulfanyl moiety is antiperiplanar with a torsion angle C11—C12—S1—C13 of -176.7 (2)°.

In the crystal structure of the title compound, there are a weak intermolecular hydrogen bond of one phenyl hydrogen atom towards the nitrile N atom (C8—H8···N4, Table 1) and a nitrogen-chlorine donor-acceptor interaction (Chu, *et al.*, 2001; Lommerse *et al.*, 1996; Ramasubbu, *et al.*, 1986) between the pyrimidinyl N atom and one of the chlorine atoms. The distance between Cl2 and N3 is 3.196 (5) Å which is definitively shorter than the sum of the corresponding van der Waals radii of Cl (1.75 Å) and N (1.55 Å). Moreover, this contact of N3 with Cl2 is nearly "head on" with N approaching Cl along the backside of C3—Cl2 with the N3···Cl2—C3 angle approximately linear 174.2 (1)° [symmetry code: -3/2 + *x*, 1/2 - *y*, -1/2 + *z*] (Fig. 2). These interactions loosly link the molecules into a two-dimensional assembly (Fig. 3). Face-to-face π-π stacking between the phenyl ring (C1—C6) and the pyrazol ring (C10—C12/N1/N2) in another molecule at 1/2+x, 3/2-y, 1/2+z complete the intermolecular interactions in the solid state. The centroid to centroid separation is 3.557 (2) Å and the angle between the two planes is 7.1 (1)°.

### Experimental

A mixture of 1-(2,4-dichlorophenyl)-3-dimethylamino-2-en-1-one (2 mmol) and 3-methylsulfanyl-4-cyano-5-amino-1*H*-pyrazole (2 mmol) in glacial acetic acid (15 ml) was stirred for 12 h at room temperature. Then the mixture was evaporated by rotary evaporation to remove the acetic acid, and recrystallized from a mixture of EtOH and DMF. Yield: 77%. (m.p. 475 K).

### Refinement

All H atoms were placed in calculated positions, with C—H = 0.93 or 0.96 Å, and included in the final cycles of the refinement using a riding model, with *U*_iso_(H) set to 1.2 *U*_eq_(C) for CH, and 1.5 *U*_eq_(C) for CH_3_.

### Crystal data

**Table:**

C_14_H_8_Cl_2_N_4_S	*F*(000) = 680
*M**_r_* = 335.20	*D*_x_ = 1.536 Mg m^−^^3^
Monoclinic, *P*2_1_/*n*	Mo *K*α radiation, λ = 0.71073 Å
Hall symbol: -P 2yn	Cell parameters from 970 reflections
*a* = 8.230 (2) Å	θ = 2.8–26.3°
*b* = 14.656 (4) Å	µ = 0.59 mm^−^^1^
*c* = 12.667 (4) Å	*T* = 293 K
β = 108.460 (5)°	Prism, colorless
*V* = 1449.3 (7) Å^3^	0.32 × 0.26 × 0.22 mm
*Z* = 4	

### Data collection

**Table:**

Bruker SMART CCD area-detector diffractometer	2965 independent reflections
Radiation source: fine-focus sealed tube	2181 reflections with *I* > 2σ(*I*)
graphite	*R*_int_ = 0.032
φ and ω scans	θ_max_ = 26.4°, θ_min_ = 2.6°
Absorption correction: multi-scan (*SADABS*; Sheldrick, 1996)	*h* = −6→10
*T*_min_ = 0.814, *T*_max_ = 0.879	*k* = −16→18
8252 measured reflections	*l* = −15→14

### Refinement

**Table:**

Refinement on *F*^2^	Primary atom site location: structure-invariant direct methods
Least-squares matrix: full	Secondary atom site location: difference Fourier map
*R*[*F*^2^ > 2σ(*F*^2^)] = 0.043	Hydrogen site location: inferred from neighbouring sites
*wR*(*F*^2^) = 0.095	H-atom parameters constrained
*S* = 1.04	*w* = 1/[σ^2^(*F*_o_^2^) + (0.0395*P*)^2^ + 0.5056*P*] where *P* = (*F*_o_^2^ + 2*F*_c_^2^)/3
2965 reflections	(Δ/σ)_max_ = 0.001
191 parameters	Δρ_max_ = 0.22 e Å^−^^3^
0 restraints	Δρ_min_ = −0.29 e Å^−^^3^

### Special details

**Table:**

Geometry. All e.s.d.'s (except the e.s.d. in the dihedral angle between two l.s. planes) are estimated using the full covariance matrix. The cell e.s.d.'s are taken into account individually in the estimation of e.s.d.'s in distances, angles and torsion angles; correlations between e.s.d.'s in cell parameters are only used when they are defined by crystal symmetry. An approximate (isotropic) treatment of cell e.s.d.'s is used for estimating e.s.d.'s involving l.s. planes.
Refinement. Refinement of *F*^2^ against ALL reflections. The weighted *R*-factor *wR* and goodness of fit *S* are based on *F*^2^, conventional *R*-factors *R* are based on *F*, with *F* set to zero for negative *F*^2^. The threshold expression of *F*^2^ > σ(*F*^2^) is used only for calculating *R*-factors(gt) *etc*. and is not relevant to the choice of reflections for refinement. *R*-factors based on *F*^2^ are statistically about twice as large as those based on *F*, and *R*- factors based on ALL data will be even larger.

### Fractional atomic coordinates and isotropic or equivalent isotropic displacement parameters (Å^2^)

**Table:**

	*x*	*y*	*z*	*U*_iso_*/*U*_eq_	
S1	0.07522 (9)	1.04589 (4)	0.33961 (6)	0.0515 (2)	
Cl1	0.27654 (9)	0.56767 (4)	0.47097 (6)	0.0587 (2)	
Cl2	0.81796 (7)	0.72025 (5)	0.77238 (6)	0.0570 (2)	
N1	0.1176 (2)	0.88388 (12)	0.44665 (15)	0.0337 (4)	
N2	0.0148 (2)	0.81205 (12)	0.45471 (14)	0.0304 (4)	
N4	−0.4254 (3)	0.99138 (17)	0.2170 (2)	0.0694 (8)	
C1	0.3585 (3)	0.65420 (15)	0.56647 (18)	0.0351 (5)	
C2	0.5304 (3)	0.65097 (16)	0.62770 (19)	0.0399 (6)	
H2	0.5982	0.6025	0.6193	0.048*	
C3	0.6000 (3)	0.72040 (16)	0.70121 (19)	0.0373 (5)	
C4	0.4996 (3)	0.79158 (16)	0.71666 (19)	0.0385 (5)	
H4	0.5468	0.8373	0.7681	0.046*	
C5	0.3289 (3)	0.79383 (16)	0.65490 (19)	0.0383 (5)	
H5	0.2616	0.8420	0.6648	0.046*	
C6	0.2541 (3)	0.72596 (14)	0.57789 (18)	0.0317 (5)	
C7	0.0708 (3)	0.73292 (15)	0.51216 (18)	0.0335 (5)	
C8	−0.0531 (3)	0.66876 (16)	0.5045 (2)	0.0437 (6)	
H8	−0.0243	0.6132	0.5411	0.052*	
C9	−0.2230 (3)	0.68669 (18)	0.4416 (2)	0.0479 (6)	
H9	−0.3036	0.6412	0.4376	0.057*	
N3	−0.2771 (2)	0.76324 (14)	0.38782 (17)	0.0427 (5)	
C10	−0.1555 (3)	0.82588 (15)	0.39444 (18)	0.0336 (5)	
C11	−0.1611 (3)	0.91130 (15)	0.34518 (18)	0.0352 (5)	
C12	0.0087 (3)	0.94280 (15)	0.38006 (18)	0.0342 (5)	
C13	0.3024 (4)	1.0381 (2)	0.4068 (3)	0.0697 (9)	
H13A	0.3249	1.0229	0.4838	0.105*	
H13B	0.3547	1.0957	0.4012	0.105*	
H13C	0.3489	0.9916	0.3714	0.105*	
C14	−0.3084 (3)	0.95536 (16)	0.2741 (2)	0.0429 (6)	

### Atomic displacement parameters (Å^2^)

**Table:**

	*U*^11^	*U*^22^	*U*^33^	*U*^12^	*U*^13^	*U*^23^
S1	0.0594 (4)	0.0374 (4)	0.0588 (4)	0.0004 (3)	0.0206 (3)	0.0114 (3)
Cl1	0.0602 (4)	0.0408 (4)	0.0615 (4)	0.0086 (3)	−0.0002 (3)	−0.0164 (3)
Cl2	0.0290 (3)	0.0678 (5)	0.0654 (5)	0.0002 (3)	0.0025 (3)	0.0056 (4)
N1	0.0313 (10)	0.0320 (10)	0.0379 (11)	0.0008 (8)	0.0110 (8)	0.0017 (8)
N2	0.0266 (9)	0.0312 (10)	0.0319 (10)	0.0020 (7)	0.0069 (7)	0.0012 (8)
N4	0.0641 (15)	0.0544 (15)	0.0648 (16)	0.0238 (12)	−0.0150 (13)	−0.0040 (12)
C1	0.0396 (13)	0.0294 (11)	0.0335 (12)	0.0021 (10)	0.0075 (10)	0.0003 (10)
C2	0.0361 (13)	0.0379 (13)	0.0445 (14)	0.0101 (10)	0.0113 (11)	0.0052 (11)
C3	0.0276 (11)	0.0421 (13)	0.0400 (13)	−0.0003 (10)	0.0075 (10)	0.0083 (11)
C4	0.0385 (13)	0.0377 (13)	0.0347 (12)	−0.0040 (10)	0.0049 (10)	−0.0013 (10)
C5	0.0391 (13)	0.0346 (13)	0.0388 (13)	0.0072 (10)	0.0089 (10)	−0.0002 (10)
C6	0.0309 (11)	0.0302 (11)	0.0312 (11)	0.0028 (9)	0.0056 (9)	0.0034 (9)
C7	0.0338 (12)	0.0325 (12)	0.0323 (12)	0.0048 (9)	0.0079 (10)	0.0029 (9)
C8	0.0417 (14)	0.0374 (14)	0.0475 (15)	−0.0015 (11)	0.0075 (11)	0.0110 (11)
C9	0.0385 (14)	0.0461 (15)	0.0550 (16)	−0.0102 (11)	0.0089 (12)	0.0053 (12)
N3	0.0289 (10)	0.0456 (12)	0.0497 (12)	−0.0016 (9)	0.0070 (9)	0.0027 (10)
C10	0.0280 (11)	0.0383 (13)	0.0327 (12)	0.0045 (10)	0.0069 (9)	−0.0005 (10)
C11	0.0353 (12)	0.0347 (12)	0.0319 (12)	0.0072 (10)	0.0054 (9)	0.0002 (10)
C12	0.0391 (12)	0.0320 (12)	0.0319 (12)	0.0021 (10)	0.0117 (10)	0.0001 (10)
C13	0.0535 (17)	0.0595 (19)	0.104 (3)	−0.0107 (14)	0.0367 (17)	0.0076 (18)
C14	0.0450 (14)	0.0362 (13)	0.0393 (13)	0.0077 (11)	0.0016 (11)	−0.0040 (11)

### Geometric parameters (Å, °)

**Table:**

S1—C12	1.739 (2)	C5—C6	1.393 (3)
S1—C13	1.796 (3)	C5—H5	0.9300
Cl1—C1	1.735 (2)	C6—C7	1.478 (3)
Cl2—C3	1.734 (2)	C7—C8	1.368 (3)
N1—C12	1.335 (3)	C8—C9	1.398 (3)
N1—N2	1.375 (2)	C8—H8	0.9300
N2—C7	1.369 (3)	C9—N3	1.315 (3)
N2—C10	1.383 (3)	C9—H9	0.9300
N4—C14	1.135 (3)	N3—C10	1.341 (3)
C1—C2	1.382 (3)	C10—C11	1.393 (3)
C1—C6	1.394 (3)	C11—C12	1.404 (3)
C2—C3	1.376 (3)	C11—C14	1.416 (3)
C2—H2	0.9300	C13—H13A	0.9600
C3—C4	1.383 (3)	C13—H13B	0.9600
C4—C5	1.375 (3)	C13—H13C	0.9600
C4—H4	0.9300		
			
C12—S1—C13	100.53 (12)	N2—C7—C6	118.02 (18)
C12—N1—N2	103.65 (17)	C7—C8—C9	120.0 (2)
C7—N2—N1	125.20 (17)	C7—C8—H8	120.0
C7—N2—C10	121.97 (18)	C9—C8—H8	120.0
N1—N2—C10	112.79 (17)	N3—C9—C8	124.7 (2)
C2—C1—C6	121.5 (2)	N3—C9—H9	117.6
C2—C1—Cl1	117.98 (17)	C8—C9—H9	117.6
C6—C1—Cl1	120.48 (17)	C9—N3—C10	115.34 (19)
C3—C2—C1	119.2 (2)	N3—C10—N2	122.7 (2)
C3—C2—H2	120.4	N3—C10—C11	132.0 (2)
C1—C2—H2	120.4	N2—C10—C11	105.25 (18)
C2—C3—C4	120.9 (2)	C10—C11—C12	105.43 (18)
C2—C3—Cl2	119.44 (18)	C10—C11—C14	126.5 (2)
C4—C3—Cl2	119.59 (18)	C12—C11—C14	128.1 (2)
C5—C4—C3	119.1 (2)	N1—C12—C11	112.9 (2)
C5—C4—H4	120.4	N1—C12—S1	122.49 (17)
C3—C4—H4	120.4	C11—C12—S1	124.61 (17)
C4—C5—C6	121.8 (2)	S1—C13—H13A	109.5
C4—C5—H5	119.1	S1—C13—H13B	109.5
C6—C5—H5	119.1	H13A—C13—H13B	109.5
C5—C6—C1	117.5 (2)	S1—C13—H13C	109.5
C5—C6—C7	119.43 (19)	H13A—C13—H13C	109.5
C1—C6—C7	123.12 (19)	H13B—C13—H13C	109.5
C8—C7—N2	115.24 (19)	N4—C14—C11	179.3 (3)
C8—C7—C6	126.7 (2)		
			
C12—N1—N2—C7	177.7 (2)	N2—C7—C8—C9	−0.3 (3)
C12—N1—N2—C10	−0.1 (2)	C6—C7—C8—C9	177.8 (2)
C6—C1—C2—C3	−0.3 (3)	C7—C8—C9—N3	−0.8 (4)
Cl1—C1—C2—C3	177.75 (18)	C8—C9—N3—C10	1.3 (4)
C1—C2—C3—C4	1.8 (4)	C9—N3—C10—N2	−0.9 (3)
C1—C2—C3—Cl2	−176.31 (18)	C9—N3—C10—C11	176.2 (2)
C2—C3—C4—C5	−1.9 (4)	C7—N2—C10—N3	−0.1 (3)
Cl2—C3—C4—C5	176.16 (18)	N1—N2—C10—N3	177.8 (2)
C3—C4—C5—C6	0.6 (4)	C7—N2—C10—C11	−177.85 (19)
C4—C5—C6—C1	0.9 (3)	N1—N2—C10—C11	0.1 (2)
C4—C5—C6—C7	−178.7 (2)	N3—C10—C11—C12	−177.4 (2)
C2—C1—C6—C5	−1.0 (3)	N2—C10—C11—C12	0.0 (2)
Cl1—C1—C6—C5	−179.02 (18)	N3—C10—C11—C14	1.9 (4)
C2—C1—C6—C7	178.6 (2)	N2—C10—C11—C14	179.4 (2)
Cl1—C1—C6—C7	0.6 (3)	N2—N1—C12—C11	0.1 (2)
N1—N2—C7—C8	−177.0 (2)	N2—N1—C12—S1	−178.37 (15)
C10—N2—C7—C8	0.7 (3)	C10—C11—C12—N1	−0.1 (3)
N1—N2—C7—C6	4.8 (3)	C14—C11—C12—N1	−179.5 (2)
C10—N2—C7—C6	−177.55 (19)	C10—C11—C12—S1	178.38 (17)
C5—C6—C7—C8	−125.1 (3)	C14—C11—C12—S1	−1.0 (3)
C1—C6—C7—C8	55.3 (3)	C13—S1—C12—N1	1.7 (2)
C5—C6—C7—N2	52.9 (3)	C13—S1—C12—C11	−176.7 (2)
C1—C6—C7—N2	−126.7 (2)		

### Hydrogen-bond geometry (Å, °)

**Table:**

*D*—H···*A*	*D*—H	H···*A*	*D*···*A*	*D*—H···*A*
C8—H8···N4^i^	0.93	2.61	3.474 (3)	154

Symmetry codes: (i) *x*+1/2, −*y*+3/2, *z*+1/2.

## References

[bb1] Allen, F. H., Kennard, O., Watson, D. G., Brammer, L., Orpen, A. G. & Taylor, R. (1987). *J. Chem. Soc. Perkin Trans. 2*, pp. S1–19.

[bb2] Bruker (1998). *SMART* Bruker AXS Inc., Madison, Wisconsin, USA.

[bb3] Bruker (1999). *SAINT* Bruker AXS Inc., Madison, Wisconsin, USA.

[bb4] Chu, Q., Wang, Z., Huang, Q., Yan, C. & Zhu, S. (2001). *J. Am. Chem. Soc.* **123**, 11069–11070. 10.1021/ja015786v 11686715

[bb5] El-Taweei, F. M. A. A. & Elangdi, M. H. (2001). *J. Heterocycl. Chem.* **38**, 981–984.

[bb6] Hernandez, S., Sanmantin, R., Tellitu, I. & Dominguez, E. (2003). *Org. Lett.* **5**, 1095–1098. 10.1021/ol034148+ 12659582

[bb7] Li, J. J., Anderson, D., Burton, E. G. & Cogburn, J. N. (1995). *J. Med. Chem.* **38**, 4570–4578. 10.1021/jm00022a023 7473585

[bb8] Lommerse, J. P. M., Stone, A. J., Taylor, R. & Allen, F. H. (1996). *J. Am. Chem. Soc.* **118**, 3108–3116.

[bb9] Olivera, R., SanMartin, R., Tellitu, I. & Dominguez, E. (2000). *Tetrahedron Lett.* **41**, 4353–4356.

[bb10] Ramasubbu, N., Parthasarathy, R. & Murray-Rust, P. (1986). *J. Am. Chem. Soc.* **108**, 4308–4314.

[bb11] Sheldrick, G. M. (1996). *SADABS*, University of Göttingen, Germany.

[bb12] Sheldrick, G. M. (2008). *Acta Cryst.* A**64**, 112–122. 10.1107/S0108767307043930 18156677

